# Spontaneously occurring melanoma in animals and their relevance to human melanoma

**DOI:** 10.1002/path.5505

**Published:** 2020-07-31

**Authors:** Louise van der Weyden, Thomas Brenn, E Elizabeth Patton, Geoffrey A Wood, David J Adams

**Affiliations:** ^1^ Wellcome Sanger Institute Wellcome Genome Campus Cambridge UK; ^2^ Arnie Charbonneau Cancer Institute University of Calgary Calgary AL Canada; ^3^ MRC Human Genetics Unit The MRC Institute of Genetics and Molecular Medicine, The University of Edinburgh, Western General Hospital Edinburgh UK; ^4^ Ontario Veterinary College University of Guelph Guelph ON Canada

**Keywords:** melanoma, naturally occurring, UV‐induced, animal model, fish, pig, cat, horse, dog

## Abstract

In contrast to other cancer types, melanoma incidence has been increasing over the last 50 years, and while it still represents less than 5% of all cutaneous malignancies, melanoma accounts for the majority of skin cancer deaths, due to its propensity to metastasise. Whilst melanoma most commonly affects the skin, it can also arise in mucosal surfaces, the eye, and the brain. For new therapies to be developed, a better understanding of the genetic landscape, signalling pathways, and tumour–microenvironmental interactions is needed. This is where animal models are of critical importance. The mouse is the foremost used model of human melanoma. Arguably this is due to its plethora of benefits as a laboratory animal; however, it is important to note that unlike humans, melanocytes are not present at the dermal–epidermal junction in mice and mice do not develop melanoma without genetic manipulation. In contrast, there are numerous reports of animals that spontaneously develop melanoma, ranging from sharks and parrots to hippos and monkeys. In addition, several domesticated and laboratory‐bred animals spontaneously develop melanoma or UV‐induced melanoma, specifically, fish, opossums, pigs, horses, cats, and dogs. In this review, we look at spontaneously occurring animal ‘models’ of melanoma and discuss their relevance to the different types of melanoma found in humans. © 2020 The Authors. The *Journal of Pathology* published by John Wiley & Sons Ltd on behalf of Pathological Society of Great Britain and Ireland..

## Introduction

**Figure 1:**
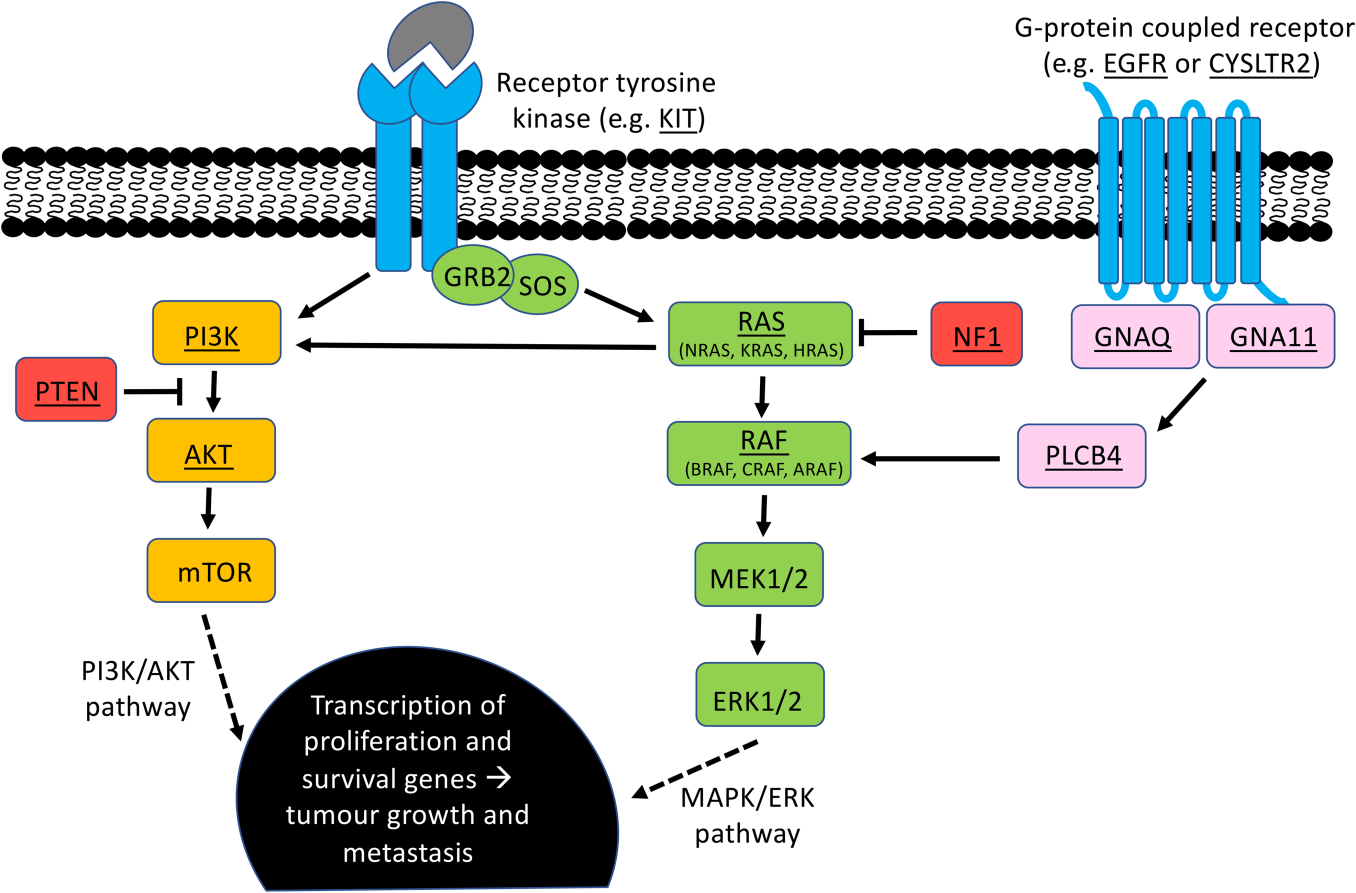
The two major signalling pathways implicated in melanoma. Commonly mutated genes are underlined. Receptors are shown in blue; proteins in the PI3K/AKT pathway in yellow; proteins in the MAPK/ERK pathway in green; proteins in the GNAQ/GNA11 pathway are pink; and proteins that have inhibitory roles in these pathways are red.

Melanoma is a tumour that arises from uncontrolled proliferation of melanocytes (pigment‐producing cells). Although the most common form of melanoma is cutaneous, it can also arise from melanocytes in the mucosal surfaces (mucosal melanoma), the eye (ocular melanoma), and leptomeninges (leptomeningeal melanoma). Although all subtypes are derived from melanocytes and thus share the same embryonic origin and cellular function, the aetiopathogenesis and biological behaviour of these melanoma subtypes are very different, with distinct landscapes of genetic alterations and different metastatic routes. The major signalling pathways implicated in melanoma are shown in Figure 1 and the most commonly mutated genes for each melanoma subtype are shown in Table 1.

**Table 1:** Summary of key genetic alterations of the different melanoma classes.

Sun status:	High CSD	Low CSD	Sun‐protected areas			
Tissue:	Cutaneous		Acral	Mucosal	Uveal	CNS
Oncogene activation	*NRA*S *BRAF* * *KIT*	*BRAF* *NRAS*	*BRAF* *NRAS* *KIT* *CCND1* (amp)	*NRAS* *KIT* *BRAF* *SF3B1* *CDK4* (amp) *CCND1* (amp)	*GNAQ* *GNA11* *CYSLTR2* *PLCB4* *SF3B1*	*GNAQ* *GNA11* *CYSLTR2* *PLCB4* *SF3B1* *NRAS* ^†^
Tumour suppressor gene loss	*NF1* *TP53* *CDKN2A* *PTEN*	*TP53* *CDKN2A* *PTEN*	*NF1* *CDKN2A*	*NF1* *PTEN* *TP53*	*BAP1*	*BAP1*
Others	*TERT* (prom)	*TERT* (prom)	*TERT* (amp)	*TERT* (amp)	*EIF1AX*	*EIF1AX*

amp, amplification; CNS, central nervous system; CSD, cumulative sun damage; prom, promoter mutations.

* The *BRAF* mutation is not the typical *V600E* variant.

^†^ There is a strong link to *NRAS* mutations in cases of childhood melanomas and neurocutaneous melanocytosis.

### Melanoma developing on sun‐exposed skin

The current fourth edition of the World Health Organisation (WHO) classification of skin tumours separates melanocytic tumours according to sun exposure: melanoma arising in intermittently sun‐exposed skin, melanoma arising in chronically sun‐exposed skin, and melanoma arising in sun‐protected skin [1]. Chronic and intermittent ultraviolet (UV) light exposures are a major cause of melanoma development in sun‐exposed skin, and UV‐induced melanoma is the most common type of melanoma in pale/fair skin individuals (people of European descent), typically occurring at 30–60 years of age (or 70 years of age in cumulative sun‐induced damage cases) [2]. The degree of cumulative sun exposure also influences the histological subtypes of melanoma. Melanomas on chronically sun‐exposed skin are typically of the lentigo maligna or desmoplastic variants, while melanoma on intermittently sun‐exposed skin is most frequently of the superficial spreading or nodular subtypes. Although cutaneous melanoma represents less than 5% of all skin malignancies, it accounts for the majority of skin cancer deaths [3], due to its aggressive nature and propensity to metastasise. Whilst the prognosis for patients with advanced‐stage melanoma has undoubtedly improved in recent years, with a current 5‐year survival of 52% and a median survival of > 60 months for those treated with combination immunotherapy (ipilimumab/nivolumab) [4], more progress needs to be made.

### Melanoma developing on sun‐protected areas

The group of melanomas developing on sun‐protected skin includes acral melanoma, genital and mucosal melanoma, ocular melanoma, and leptomeningeal melanoma. Other rare subtypes of melanoma that arise independent of UV light exposure are Spitz melanoma, melanoma arising in blue naevi, and melanoma arising in congenital naevi.

#### Acral melanoma

Acral melanoma arises on sun‐protected sites, including the palms of the hands, soles of the feet, and the nail apparatus (subungal) of the middle‐aged and elderly, with a mean of around 60 years of age. Although the absolute incidence is independent of ethnicity, the relative incidence is highest in individuals with darker skin types (African, Middle Eastern, Latin American or Asian ethnicity) [2]. The majority of melanomas are of the acral lentiginous or nodular subtype. Acral melanoma carries a particularly poor prognosis as it is often detected at a more advanced stage.

#### Genital and mucosal melanoma

In comparison to cutaneous melanoma, genital and mucosal melanomas (MMs) are much rarer, representing only 0.8–3.7% of all melanomas [5]. They are relatively more common in Asian and black populations, where they account for 9–22% of all melanoma cases [5]. This is due to the rarity of UV light‐associated melanoma subtypes in this population. Like acral melanomas, genital and MMs typically occur at 60 years of age [2]. The majority of MMs present in the nasal cavity and the accessory sinuses, the oral cavity, anorectum, vulva, and vagina, but they can arise in almost every mucosal membrane, including the parotid glands, oesophagus, and middle ear [6]. Histologically, they are most commonly of the lentiginous mucosal subtype, followed by nodular melanoma. MMs are known to behave more aggressively with a less favourable prognosis compared with other melanoma subtypes. Most MMs occur in occult sites, which together with the lack of early and specific signs, contributes to late diagnosis and poor prognosis, with a 5‐year survival rate of 25–33%, according to disease stage and location [7]; metastasis to the lungs and liver occurs in 50% of cases with head and neck melanoma [7].

#### Ocular melanoma

Ocular melanoma (OM) is the most common primary cancer affecting the eye, and is classified based on the anatomic site of origin: conjunctival melanoma or uveal melanoma (UM). The majority of OMs originate from the uvea (95%), comprising the posterior uvea (choroid 90% and ciliary body 5%) and the anterior uvea (iris 5%). There is a wide age distribution for UM, with an average age of 60 years [2]. Although the primary tumour can be effectively surgically removed, half of all patients develop metastasis (typically the liver or lung) and these patients have a poor prognosis, with a median survival time of 4–15 months [8]. Conjunctival melanoma generally presents as a pigmented nodular lesion typically on the bulbar conjunctiva and often involves the limbus [9]. Approximately 20–30% of conjunctival patients develop metastatic disease, either directly extending into the eyeball and orbit or as more distant metastases to the lungs, brain, liver, skin, bones, and gastrointestinal tract [10].

#### Primary melanocytic neoplasms of the central nervous system

Primary melanocytic neoplasms of the central nervous system (CNS), which can be divided into either benign or malignant and localised or diffuse, are presumed to arise from melanocytes in the leptomeningeal tissue [11]. However, primary melanoma of the CNS is rare, with an incidence of 0.005 per million people [12] (compared with 2.8–3.1 per 100 000 for cutaneous melanoma [13]), whereas secondary intracranial melanomas are more common, with ~10–40% of melanomas metastasising to the CNS [14]. Meningeal melanocytomas are solitary, low‐grade tumours with no invasion of surrounding structures, with a reported annual incidence of 1 case per 10 million population [15]. In contrast, meningeal melanomas are solitary, highly aggressive, radioresistant tumours that can metastasise, resulting in poor prognosis, with a reported annual incidence of 0.5 cases per 10 million population [12]. The diffuse presenting tumours (generally involving large expanses of the subarachnoid space) are meningeal melanocytosis, which is a proliferation of cytologically bland melanocytic cells, and meningeal melanomatosis, which is a primary melanoma of the CNS. Diffuse meningeal melanocytic tumours are rare and as such, population‐based incidence is not available; however, they are strongly linked with neurocutaneous melanocytosis, which is a rare disease that presents before 2 years of age and is typically associated with giant congenital naevi [16]. The prognosis for diffuse meningeal melanocytic tumours, in the setting of neurocutaneous melanocytosis, is extremely poor, with no known effective treatment options and no reports of survivors in the literature [17].

#### Melanoma of unknown primary

Melanoma of unknown primary (MUP) refers to metastatic melanoma occurring in lymph nodes, subcutaneous tissue, or visceral sites in the absence of a detectable primary tumour [18], and accounts for ~2–9% of metastatic melanomas [19]. The most common clinical presentation of MUP is lymph node disease without clinical or radiological evidence of visceral involvement [18] and typically presents in the fifth and sixth decades of life [20]. The origin of MUP is not fully understood and explanations have included regression of primary cells at the site of origin (regression is found in melanoma with a frequency of 10–35% [21]), misdiagnosis of the original primary cutaneous melanoma, and *de novo* malignant transformation of ectopic melanocytes in lymph nodes or other organs [18]. Interestingly, genomic analysis of MUPs has found they have a mutational profile more similar to cutaneous melanoma than that of mucosal melanoma or CNS melanoma, including mutations in *BRAF* and *NRAS* [22], suggesting that regression or misdiagnosis of a previous cutaneous lesion was the primary melanoma.

## The use of the mouse to model melanoma

In order to study basic mechanisms and pre‐clinically test novel therapies, accurate animal models are required. The most popular animal model for melanoma research is the laboratory mouse, and there are numerous mouse models of cutaneous melanoma (reviewed in [23]) and uveal melanoma (reviewed in [24]), as well as several models of mucosal melanoma [25] and leptomeningeal melanoma [26]. Although each model has its own distinct advantages and disadvantages (transgenic models, gene knockout/knock‐in models, xenograft models, or UV/carcinogen‐induced models), our understanding of the molecular mechanisms of melanoma development in humans has been greatly improved by modelling various pathways in these mice [27, 28]. However, it is worth mentioning that interpreting results from melanoma studies in mice needs to take into consideration several key aspects: (1) the heterogeneity of melanoma in humans, which would not be recapitulated in inbred laboratory mice; (2) the spontaneous development of melanoma in humans, as melanoma does not spontaneously occur in the mouse; and (3) differences in the structure of the skin between humans and mice, as melanocytes are located in the epidermis in humans and in the dermis in mice [29], meaning that there is a different melanocyte microenvironment, which may account for differences in melanoma development and progression between the two species. Thus, it is worth looking at animals that spontaneously develop melanoma (of all subtypes) and assessing what relevance they have to human melanoma as alternative animal models.

## Melanoma in the ‘wild’

**Figure 2:**
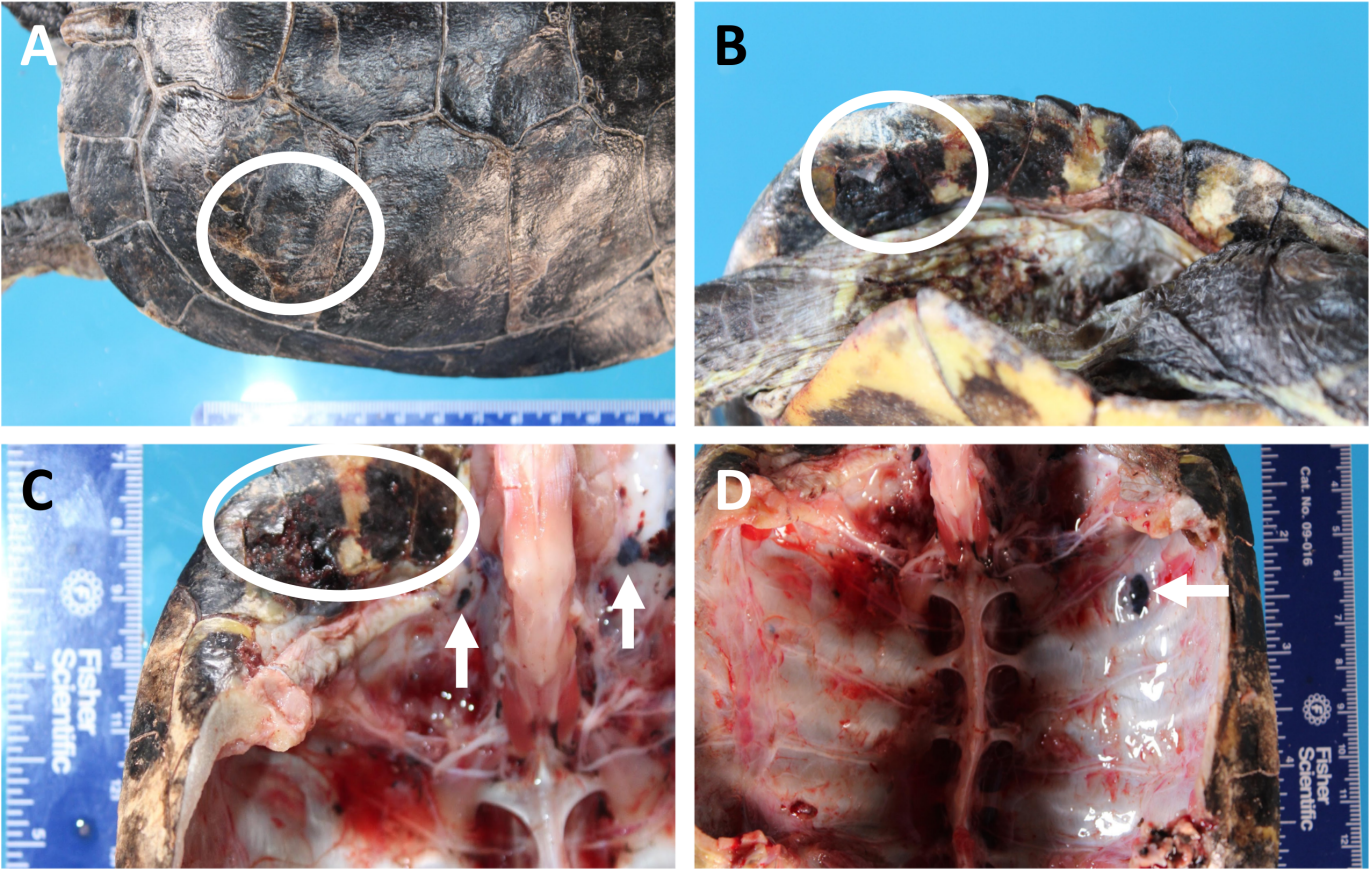
Metastatic melanophoroma in a red‐eared slider turtle. (A, B) Multifocal malignant melanophoromas on the shell (circle). (C, D) The lesions invaded through the shell (circle) into the peritoneal cavity (arrows). The photographs were kindly provided by Luke Haydock, Department of Pathobiology, University of Guelph, Guelph, Ontario, Canada.

There have been reports of melanoma spontaneously occurring in a wide range of species, including fish, reptiles, birds, and mammals, with some examples detailed in Table 2. In reptiles, tumours of pigment‐producing cells of the skin are called chromatophoromas and are subclassified on the basis of the type of pigment, specifically melanophoromas or iridophoromas. A review of chromatophoromas that arose in 26 reptiles (6 snakes, 19 lizards, and 1 tortoise) found that six of the 20 melanophoromas were classified as malignant, due to the presence of intravascular tumour cells, visceral metastases, high pleomorphism, and/or mitotic figures [43]. Microscopically, most of the tumours were composed of spindle cells with varying pigmentation, and both melan A and S100 were expressed by all of the tumours by immunohistochemistry [43]. Six of the nine reptiles that were euthanised immediately after diagnosis or following tumour recurrence had visceral metastases [43]. Figure 2 shows a metastatic melanophoroma found at autopsy of a 60‐year old red*‐*eared slider turtle (*Trachemys scripta elegans*).

**Table 2:** Some examples of the wide range of species in which spontaneously occurring melanoma or melanocytoma has been reported.

Animal	Clinical presentation	Diagnosis and histopathology report	Outcome	Ref
Nurse shark (*Ginglymostoma cirratum*)	A 5.5‐year history of a 6‐cm black, raised nodular skin lesion located on the right side of the proximal tail	**Cutaneous melanoma.** The biopsy comprised interlacing streams of neoplastic spindle cells with scant eosinophilic cytoplasm and vesicular nuclei with prominent nucleoli. A few of the cells contained melanin. No vascular invasion was noted	Euthanised for systemic illness approximately 4.5 months after diagnosis (although no evidence of metastasis was found on histopathologic evaluation of the skin and viscera)	30
Haller's round ray (*Urobatis halleri*)	Presented with multiple black raised nodular masses on the dorsal surface	**Cutaneous malignant melanoma.** The masses were composed of proliferative sheets of melanocytes exhibiting mild anisocytosis and anisokaryosis	Approximately 2 months following the biopsy, the ray became acutely anorexic and was found dead	31
Coral trout (*Plectropomus leopardus*)	20/136 of line‐caught coral trout from two locations in the Great Barrier Reef Marine Park (in 2010–2012) showed a dark growth lesion, covering <10% of body surface to almost complete coverage	**Melanosis and melanoma.** The lesions showed a tumourous appearance of disorganised pleomorphic cells containing melanosomes, consistent with melanophorous‐macromelanophorous polymorphic melanoma (MMPM)	N/A	32
Florida pine snake (*Elaphe obsoleta rossalleni*)	Presented with a black dermal tumour, dorso‐laterally in the posterior third of the body	**Malignant melanoma.** The melanotic cells were polyhedral and uniformly round or nearly round. The vesicular nuclei occupied most of the cellular space and the nucleoli were prominent. The scanty amount of protoplasm was heavily impregnated with melanin granules. Widespread metastasis	Two months after tumour excision, further small black tumours appeared in various parts of the skin and the general condition of the snake began to deteriorate; it died soon after	33
Mandarin duck (*Aix galericulata*)	Presented with several small multinodular masses on the dorsal surface of the upper bill	**Metastatic malignant melanoma.** The tissue consisted of multiple nests and lobules of neoplastic melanocytes (with prominent areas of necrosis). The lymphoid tissues contained nests and individual neoplastic melanocytes	Two months later, the tumour had enlarged considerably. The duck developed severe dyspnea and was euthanised	34
Macaroni penguin (*Eudyptes chrysolophus*)	Presented with a caseous and necrotic mass that engulfed the upper beak and extended into the rostra sinuses	**Malignant melanoma.** The tissue contained an infiltrative, deeply pigmented melanocytic neoplasm that extended from superficial to deep dermal regions and had effaced normal tissue architecture	The penguin exhibited dyspnea as a result of invasion of the tumour into the rostral sinuses, and was immediately euthanised	35
Rabbit (*Oryctolagus cuniculus*)	Presented with an irregular black mass (4 × 3 × 2 cm) on the skin of scrotum, with ulceration and bleeding	**Cutaneous malignant melanoma.** The tumour cells were positive for melanocyte markers (HMB‐45, PNL2, melan A, and S100) and Ki‐67	The perioperative period concluded with no problems; however, despite subsequent clinical progress, the rabbit died after 2 weeks due to metastasis	36
Pygora goat (*Capra aegagrus hircus*)	Presented with a recurrent ulcerated, black‐pigmented, 2.5‐cm mass at the base of the left horn	**Malignant melanoma.** The masses consisted of moderately pleomorphic, polyhedral to spindle cells containing variable amounts of dark brown intracytoplasmic pigment granules. Multiple black foci of metastasis observed in the liver	While restraining the goat for physical examination the left horn broke off at the base with minimal force. Due to the poor condition of the goat, it was immediately euthanised	37
Huacaya alpaca (*Vicugna pacos*)	Presented with a chronic, non‐healing wound involving the left external nostril	**Metastatic mucocutaneous melanoma.** Malignant melanoma was diagnosed by histology of biopsy specimens. At post‐mortem, numerous masses present throughout the body were composed of spindloid to polygonal cells with indistinct cell borders and moderate amounts of cytoplasm containing abundant melanin	The alpaca was euthanised 10 days after the diagnosis on the basis of the poor prognosis and rapid clinical deterioration	38
Pygmy hippopotamus (*Choeropsis liberiensis*)	Presented with multiple raised and pigmented skin masses	**Dermal malignant melanoma and concurrent melanocytoma.** Initial impression smears of one ulcerated lesion were consistent with inflammation; however, subsequent histopathological findings from a skin biopsy revealed an underlying malignant melanoma	There was no sign of recurrence 34 months post‐surgery (no lymph node involvement)	39
African lion (*Panthera leo*)	Presented with a 4‐month history of left maxillary lip swelling. Examination showed a pigmented mass at the level of the left maxillary canine tooth	**Dermal malignant melanoma.** Histopathologic evaluation of the biopsies revealed a malignant dermal melanoma with no evidence of metastasis	The lion received radiotherapy and immunotherapy treatments	40
Aberdeen Angus cow (*Bos taurus*)	Presented with a large, pedunculated cutaneous mass protruding from the left flank fold and an enlarged left pre‐femoral lymph node	**Congenital amelanotic melanoma with nodal metastasis**. Histologic examination revealed a homogeneous population of neoplastic cells staining positively for S100 and melan A	Two months later, the calf became acutely recumbent and was euthanised after clinical examination revealed widespread metastasis	41
Cynomolgus monkey (*Macaca fascicularis*)	A pigmented raised mass (2 cm diameter) at the dorsal aspect of the neck	**Cutaneous melanocytoma.** Histologically, the mass was composed of poorly demarcated, heavily pigmented melanocytes diffusely infiltrating the dermis. The neoplastic cells were fairly uniform, round‐to‐polygonal in shape, and arranged in packets, supported by a fibrovascular stroma. The nuclei were mostly round and lacked nuclear atypia	Not reported	42

However, whilst it is of interest to see the similarities of the histological descriptions of these melanomas with that seen in human melanoma, these case reports demonstrate the low frequency of the spontaneous development of this tumour type in these animals. Indeed, malignant melanomas are rare in fish and extremely rare in reptilian and avian species. In addition, the specialised habitat required for many of these animals makes them unfeasible for use as a model organism. However, there are several non‐genetically‐modified laboratory‐bred animals that develop melanoma (due to selective breeding of affected individuals), some spontaneously and others after exposure to radiation, such as specific species of fish, opossums, and pigs. In addition, there are several domesticated animals in which the spontaneous development of melanomas is relatively common, such as the dog and horse. These animals offer many important advantages as ‘models’ of melanoma in humans, which are discussed below.

## Laboratory‐bred animal models of spontaneously occurring melanoma

### Fish

**Figure 3:**
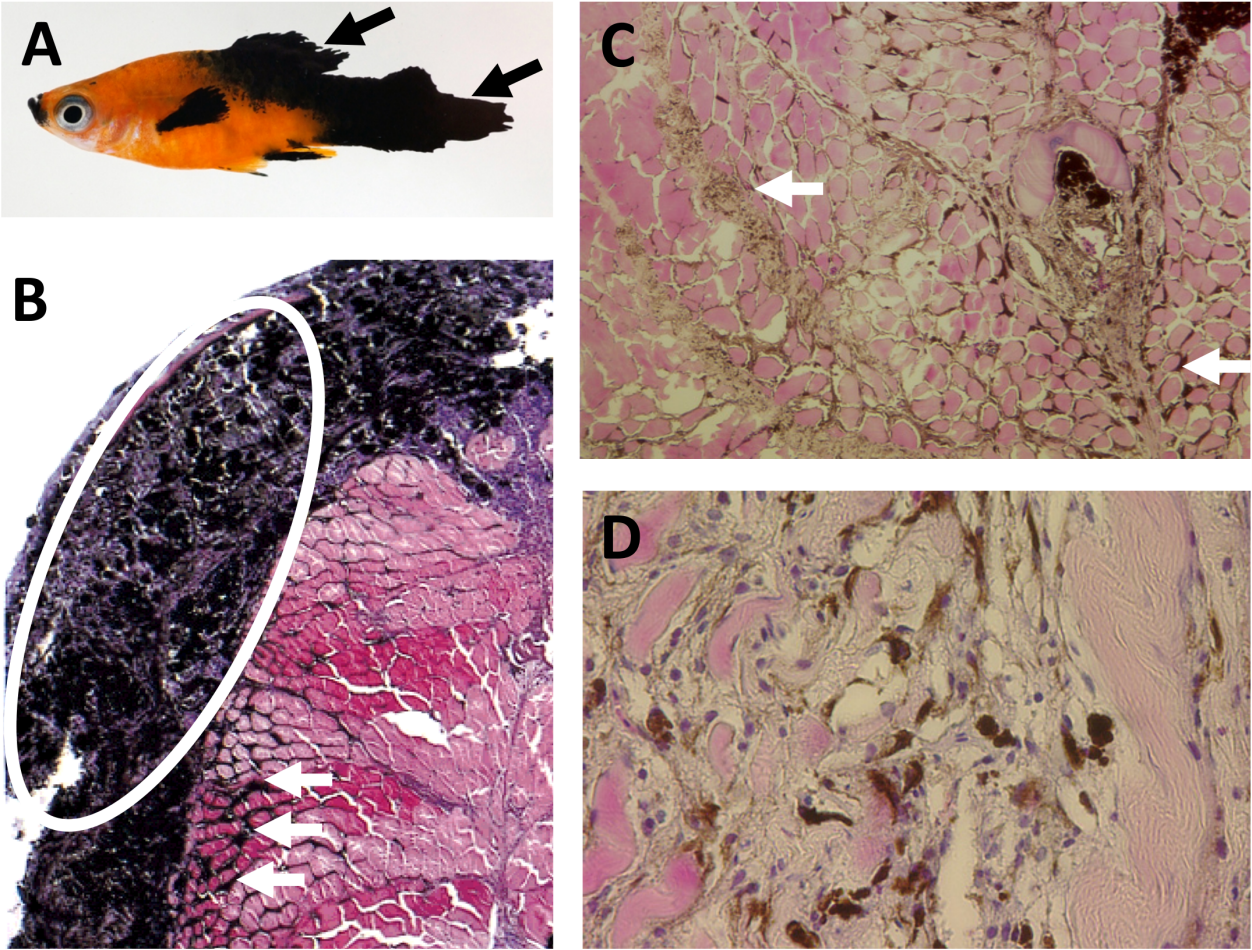
Melanoma in a *Xiphophorus*. (A) Macroscopic photograph of a platyfish/swordtail backcross hybrid (*Tu*+/−, *R*−/−) with a large pigmented melanoma in the posterior and on the pectoral fin (arrows). (B–D) Images of H&E‐stained sections of a large exophytic melanoma that covers the surface of the skin (circle), while heavily pigmented melanoma cells can be seen infiltrating the muscle (arrows) (25×, 100× and 200× orignal magnification, respectively).

The first fish models of melanoma were established in *Xiphophorus* (commonly known as platyfish or swordtails), which is a genus of euryhaline and freshwater fish native to Mexico and northern Central America. The *Xiphophorus* model (also known as ‘Gordon–Kosswig–Anders’ melanoma) was originally introduced in the late 1920s and uses interspecies platyfish that spontaneously develop melanoma in a genetically controlled manner (reviewed in [44]). The platyfish (*X. maculatus*) parental line carries the *spotted dorsal* (*Sd*) macromelanophore pigmentation pattern. These spots are non‐malignant melanocytic lesions or hyperpigmentation on the dorsal fin, and the macromelanophore pattern is considered a benign precursor to melanoma. This is controlled by the pigment‐cell‐specific oncogene locus *Tu* (for tumour), the oncogenic activity of which is suppressed by the tumour suppressor locus *R* (for regulator). In the classical cross, platyfish, which carry *Tu* and *R*, are crossed with swordtails (*X. hellerii*), which carry neither locus and thus have no pigmentation pattern. When the resulting F1 hybrids are backcrossed with swordtails, 25% of the progeny inherit the *Tu* oncogene as heterozygotes, but not the *R* locus, and these *Tu/−* hybrids develop spontaneous, highly malignant melanoma (Figure 3).

Positional cloning identified the melanoma‐inducing *Tu* locus as a novel receptor tyrosine kinase termed *Xmrk* (*Xiphophorus melanoma receptor tyrosine kinase*) [45] that originated from a tandem gene duplication of the *Xiphophorus EGFR* (*epidermal growth factor receptor*) gene, and is the fish orthologue of human EGFR. The oncogenic activity of *Xmrk* depends on its overexpression, which correlates with the aggressiveness of the melanoma, as *Xmrk* expression increases with malignancy [46]. *Xmrk* carries two activating mutations that result in a constitutively active receptor whose signals elicit a variety of cellular responses including proliferation, anti‐apoptosis, and cell motility that ultimately result in melanoma development (reviewed in [44]). Interestingly, despite being oncogenic, *Xmrk* has been maintained for several million years in the *Xiphophorus* melanoma model, and clues for the positive selection were found in experiments with *Xiphophorus cortezi* that showed females prefer males with an enhanced *Sc* pattern [47]. In addition, *Xmrk* males (regardless of *Sc* phenotype) were more aggressive than *Xmrk*‐deficient (wild‐type) males [48] and collections of males from four natural populations of these fish found that those with malignant melanomas were significantly larger than both *Xmrk* males without melanomas and *Xmrk*‐deficient males [49]. Based on genetic linkage analyses, the *Cdkn2ab* gene was proposed as the critical component of the tumour suppressor *R* locus (so named due to 55% amino acid homology with the human tumour suppressor genes *CDKN2A* and *CDKN2B* that are frequently mutated in melanoma) [50]. *Cdkn2ab* was subsequently shown to have a melanoma‐suppressive function in *Xmrk* transgenic medaka models [46], but it is not clearly established if the *R* locus is *Cdkn2ab*.

The relevance of the *Xiphophorus* model to human melanoma stems from the fact that Xmrk, being an oncogenic version of the well‐studied EGFR, uses several already known signalling pathways, including the Ras/Raf/MAPK (mitogen‐activated protein kinase) pathway that is commonly mutated in human melanomas (reviewed in [51]). This fish model significantly contributed to the understanding of the importance of the receptor tyrosine kinase (RTK)–Ras–MAPK pathway signalling in melanoma, along with engineered melanoma models in mice, cell line studies, and human chromosomal rearrangements (reviewed in [52]). Similarly, Xmrk also induces the PI3K signalling pathway, and it was the discovery of constitutive activation of STAT5 correlating with the aggressiveness of melanoma in *Xiphophorus* [53] that prompted investigation of STAT5 in human melanoma [54].

*Xiphophorus* also represents a relevant model as it is one of only a few animal models for which the induction of melanomas can occur by exposure to UV radiation alone, and studies in these fish were important for proving the role that solar UV‐B radiation plays in the formation of cutaneous melanoma [55]. More recently, gene expression profiling of melanomas produced in these fish showed that the high‐expressing *Xmrk Xiphophorus* melanomas share transcriptomic features and molecular functions of highly proliferative, dedifferentiated human melanoma [56].

### Opossum

The gray short‐tailed opossum, *Monodelphis domestica*, is a small (~100 g) pouchless marsupial native to South America that has been maintained in outbred colonies in laboratories since 1978 (reviewed in [57]). *Monodelphis* has the distinction of being the only naturally existing mammal, other than humans, known to be susceptible to melanoma in response to UV radiation alone. It was long suspected that UV radiation was involved in the aetiology of cutaneous melanoma in humans; however, it was studies in *Monodelphis* that proved that UV radiation can act as a complete carcinogen for melanoma induction, and that DNA damage is involved in melanoma formation [58]. UV (primarily UV‐B)‐exposed *Monodelphis* develop a variety of hyperplastic and neoplastic skin lesions on their shaved back, including malignant melanoma [59], and UV exposure of neonatal *Monodelphis* results in the development of naevus, with further repeated UV exposure into adulthood (up to 45 weeks of age) resulting in progression to malignant melanoma with metastases to lymph nodes [60].

Genetic characterisation of the UV‐induced melanocytic hyperplasias and melanomas of *Monodelphis* found that altered levels of expression of *CDKN2A* and *ARF* genes are associated with the aetiology of melanoma formation and progression in these animals [61]; both *CDKN2A* and *ARF* are major contributors to melanoma initiation and progression in humans [2]. In addition, *Monodelphis* has both DNA photoactivated excision repair and ubiquitous excision repair mechanisms, as do humans [62]; in contrast, an absence of photoactivation of UV‐induced pyrimidine dimers is detectable in mouse epithelial cells *in vivo* [63]. Studies in *Monodelphis* have also shown that SPF 15 sunscreen is effective in dramatically reducing the incidence of melanocytic naevi [64], and since it was demonstrated that 43% of *Monodelphis* with naevi progress to malignant melanoma in middle‐to‐late adulthood [65], this makes *Monodelphis* a model of sunscreen‐mediated melanoma prevention.

### Pig

The spontaneous occurrence of melanoma in pigs is generally very low; during a 5‐month study of pig carcasses from two abattoirs, only 220/747 014 (0.03%) had gross cutaneous and lymph node lesions suggestive of melanoma [66]. Interestingly, however, 174/176 of the cutaneous lesions submitted for histological analysis were spontaneously regressing melanomas (the remaining two were non‐regressing melanomas). Through selective breeding to increase the incidence of melanoma, three models of hereditary melanoma have been established: the Sinclair miniature swine, the Munich miniature swine Troll, and the melanoma‐bearing Libechov minipig, each showing congenital or early postnatal development of melanoma and spontaneous regression associated with depigmentation.

#### Sinclair miniature swine

The Sinclair miniature swine was derived from the Hormel miniature pig at the Hormel Institute in the USA. The first observation of cutaneous melanoma in the Sinclair miniature swine strain was in 1967, at an incidence of 21% [67]. With subsequent selective breeding, the incidence is now at ~60%, with black‐coat‐colour pigs developing multiple congenitally‐ or postnatally‐appearing skin lesions (exophytic, flat, ulcerated, locally necrotic) and red‐coat‐colour pigs developing no tumours [68].

**Figure 4:**
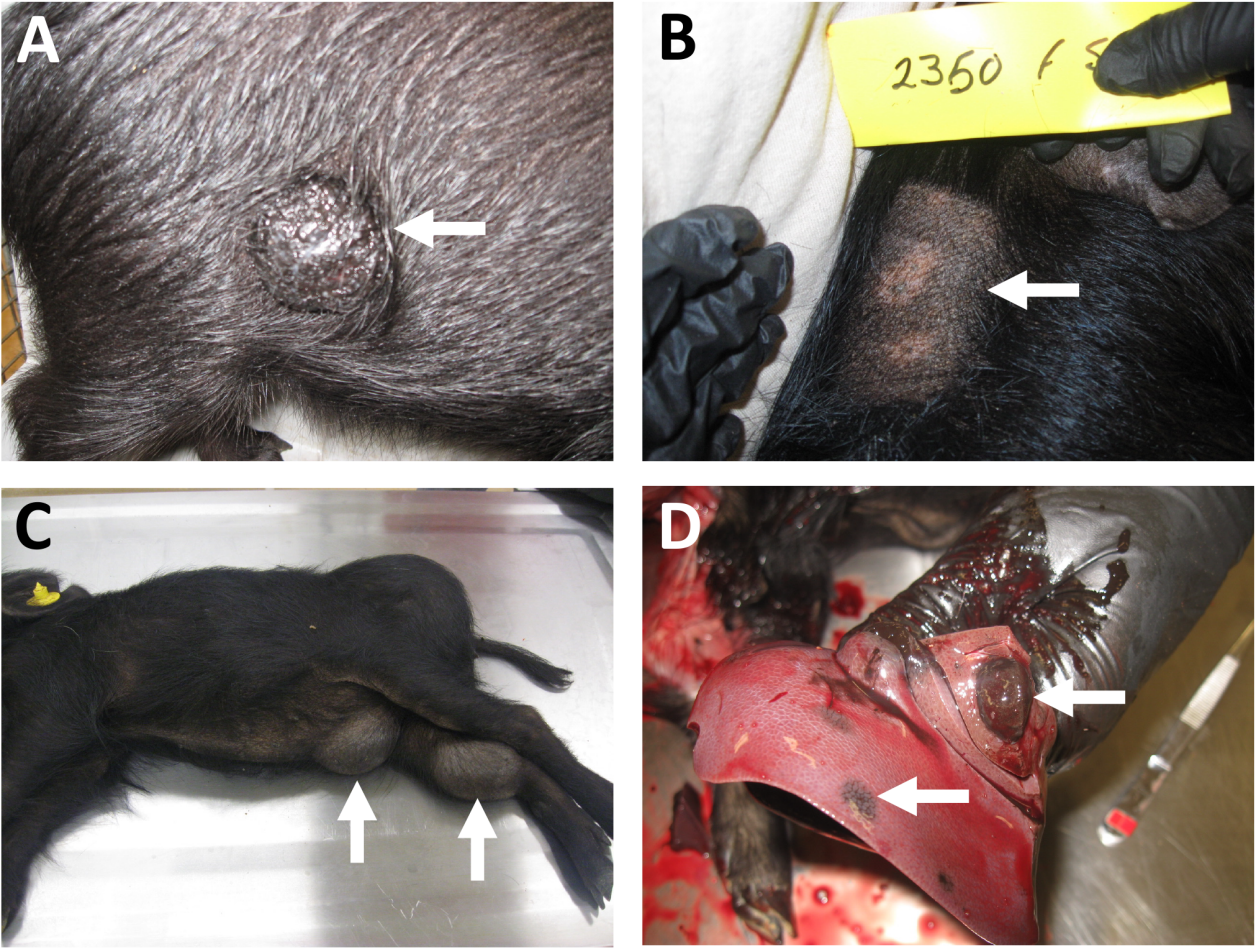
Melanomas in Sinclair miniature pigs. (A) A cutaneous melanoma on the flank (arrow). (B) A regressed cutaneous melanoma showing areas of skin depigmentation (arrow). (C) Large cutaneous melanomas on the belly and inner hind limb (arrows). (D) Hepatic metastases from a cutaneous melanoma (arrows). The photographs were kindly provided by Derek Brocksmith, Sinclair BioResources, Missouri, USA.

The Sinclair miniature swine model has numerous features in common with human melanoma. Firstly, they develop benign melanocytic lesions (benign naevi) that are capable of malignant transformation (superficial spreading melanoma or nodular melanoma) with metastatic spread of the deeply invasive tumours (to lymph nodes and visceral organs, mainly lungs and liver), analogous to the progression of melanoma seen in humans [69] (Figure 4). Secondly, these melanomas are capable of undergoing spontaneous regression, as has been reported in some human melanoma patients (Figure 4). In addition, that several melanoma lesions can progress and regress simultaneously in the same pig [70] suggests that tumour heterogeneity may play a significant role in the natural history of swine melanoma, similar to humans. The Sinclair swine melanoma model also affords the unique opportunity to perform sequential biopsies on a single lesion (due to the large size of the swine melanomas) and the opportunity to perform biopsies on progressing and regressing tumours. Finally, there is a heritable component, as seen with some melanomas in humans, although the exact genetic determinants responsible are not yet known (structural alterations in chromosomes 2, 3, 6, 7, and 12 may represent the initial step of melanoma development [71]).

Spontaneous regression of melanomas due to a tumour‐related immune response has been reported in human patients. In the Sinclair miniature swine, melanoma regression occurs in a two‐phase process, with the first phase (typically occurring at 2 months of age) characterised by a massive macrophage infiltration, followed by the second phase (typically occurring at 4 months of age) characterised by lymphocyte infiltration and complete elimination of melanomas [72]. The tumour‐infiltrating lymphocytes in the second stage were identified as cytotoxic (CD4^−^/CD8^+^) and were found at much higher levels than in the peripheral blood, suggesting that the cytotoxic T‐lymphocytes play a key role in the final elimination of the melanoma. However, as antibodies against melanoma antigens were found in the pigs with spontaneously regressing melanoma, usually preceding or appearing together with tumour regression and depigmentation, an antibody‐mediated immune response directed against common antigens presented by both malignant and normal melanocytes may also play a role [73]. Spontaneous regression has also been found to be associated with defective telomerase activity [74] and increased sensitivity of the melanoma cells to apoptosis [75].

#### Munich miniature swine Troll

The Munich miniature swine (MMS) Troll is historically the second swine model with hereditary melanoma, although it is not as widely used as the other two models. It was established at the University of Munich in Germany in 1986, using a melanoma‐bearing boar with two unaffected sows from the herd originally developed from the Hanford and the Columbian miniature swine [76]. Similar to the other pig models, selective breeding has led to a high incidence of malignant tumours (~70%). Benign melanocytic lesions are also observed, the skin lesions are already present at birth or appear within 2 months after birth, and complete spontaneous regression of melanomas accompanied by hair and skin depigmentation occurs. Interestingly, elevated expression of porcine endogenous retroviruses was detected in melanomas and pulmonary metastasis‐derived cell cultures from the MMS Troll [77], and upregulated transcription and expression of human endogenous retrovirus (HERV) group HERV‐K(HML‐2)‐encoded proteins have been reported in human melanomas [78]. However, it is not clear what role this retroviral material plays in the onset and/or promotion of tumourigenesis [79].

#### Melanoma‐bearing Libechov minipig

Generation of the melanoma‐bearing Libechov minipig model was first started in 1966 at the Czech Academy of Sciences in Libechov by importing Goettingen miniature swine from the University of Goettingen in Germany that were cross‐bred with Minnesota miniature pigs from the Hormel Foundation in the USA and Vietnamese pigs from German zoos [80]. Subsequent years of cross‐breeding with pigs of several commercial meat breeds and Vietnamese pigs (to maximise genetic variability) produced a population of more than 2,000 genetically heterogeneous descendants, with the first few black piglets with melanoma being observed in 1989. Selective breeding of melanoma‐bearing animals confirmed the genetic predisposition to melanoma, with an incidence of ~50%, and this new pig model was termed the melanoma‐bearing Libechov minipig (MeLiM) [81, 82]. Subsequent breeding programmes have increased the melanoma incidence to ~80%, with tumour management used to increase their survival and allow their use in breeding [83].

Reflecting the multi‐hybrid nature of this strain, MeLiM pigs show variability in coat colour, with the black pigs being most affected by melanoma. The black pigs develop three types of melanocytic skin lesions (superficial spreading melanoma, nodular melanoma or unclassified melanoma) and display lesions ranging from lentigo to metastatic melanomas [84]. The metastases typically occur in the lymph nodes, lungs, and spleen (with heavily affected animals bearing metastases in other visceral organs). MeLiM melanomas share a lot of similarity with human melanomas, in terms of both their biochemistry and immunohistochemical characteristics. For example, the deep black pigmentation of the tumours is due to the high concentration of melanosomes with a high proportion of melanin [85], as found in the Sinclair swine [86] and human nodular melanoma [87]. Similarly, increased expression of key proteins in melanoma diagnosis and malignancy is elevated in both MeLiM melanomas and human melanomas, such as S100, RACK1, and tenascin C (reviewed in [83]). In addition, mass spectrometry of growing MeLiM melanoma tissue showed high concentrations of zinc (due to elevated metallothionein content) [88], which has also been reported in human melanomas [89].

Similar to the Sinclair miniature swine model, the development of melanoma in the MeLiM model is heritable and polygenic. Although the precise genetic determinants are not yet known, a genome‐wide association study (GWAS) revealed several loci on chromosomes 2, 5, 7, 8, and 16 showing significant associations with melanoma occurrence and progression, and comparison to human melanoma GWAS results indicated shared association signals at *CDKAL1* and *TERT* loci as well as the proximal *CCND1*, *FTO*, *PLA2G6*, and *TMEM38B‐RAD23B* loci [90]. Further similarities with the Sinclair miniature swine model include the cutaneous melanoma lesions being present either at birth or within the first 2 months of life, and the lesions either progressing (developing extensive metastasis that leads to death within the first 3 months of life) or spontaneously regressing (with associated skin and bristle depigmentation) [83]. Spontaneous regression occurs asynchronously between the different lesions on the animal (regression is usually completed around 6–12 months of age) and the depigmentation can sometimes spread to the entire body, suggesting activation of immune cells against an antigen common to normal melanocytes as well as melanoma cells [81]. Spontaneous regression in both the Sinclair miniature swine and the MeLiM melanomas represents a promising immunological model for monitoring immune cells participating in anti‐melanoma reaction. Double‐positive (DP) T‐lymphocyte (CD4^+^/CD8^high^) populations of effector/memory T‐cells [both in the peripheral blood (PBL) and within the tumour (TIL)] have been reported to expand in MeLiM animals during melanoma regression and it is postulated that they are involved in the regression process [91]. This contrasts with the Sinclair miniature swine model, in which populations of DP lymphocytes were generally consistent in all TIL and PBL populations examined [92]. Nevertheless, a significant increase of DP lymphocytes has also been reported in human melanomas and metastases [93].

Finally, the role of the microbiome in melanoma development has been studied in MeLiM piglets. The number of *Fusobacteria* was higher in melanoma samples compared with healthy skin, and also in progressing compared with regressing melanomas [94]. Interestingly, the abundance of *Fusobacteria* in the gut is connected with colorectal cancer development and progression in humans (reviewed in [95]); thus, it will be of interest to see if the skin microbiome also plays a role in melanoma development or progression in humans.

## Domesticated animal models of spontaneously occurring melanoma

### Horse

In horses, skin tumours are the most common among neoplasms, with up to 15% of all equine skin tumours being melanocytic [96]. Although more than 90% of these are benign at initial presentation, if left untreated, up to two‐thirds can progress to overt malignancy and are able to undergo widespread metastasis [96, 97, 98]. The vast majority appear in grey or white horses, usually at or before the age of 5 years, which corresponds to when their coat colour changes [96, 97, 99, 100]. A review of the pathological characteristics of equine melanoma (based on a retrospective study of 53 cases) proposed four manifestations of equine melanotic disease: melanocytic naevus, discrete dermal melanoma (benign and malignant forms), dermal melanomatosis, and anaplastic malignant melanoma [101]. The range of intradermal naevi seen in horses resembles those seen in humans, with common melanocytic naevi, cellular blue naevi, and combined cellular blue naevi, showing histopathological features in common with their human counterparts [102]. Although discrete dermal melanoma and dermal melanomatosis were described as distinctly separate entities by Valentine [101], these are generally histologically indistinguishable, presenting as indistinct, heavily pigmented tumour cells in the deep dermis; thus, it seems probable these diagnoses exist as a progression sequence [103]. Interestingly, in contrast to melanomas in solid‐coloured horses, which, although rarer, are characterised by high malignancy and early metastases, melanomas in grey horses tend to have an extended period of benign growth prior to malignant transformation and metastasis [96, 104]. Nevertheless, grey and white horses are associated with the development of dermal melanomatosis such that beyond the age of 15 years, at least 80% of grey horses will have melanomas at some location [93]. Similar to human melanoma, equine melanomas are typically positive for S100, PCNA, HMB‐45, Ki‐67, T‐311, and CD44 by immunohistochemistry [100]. In addition, *RACK1*, which was originally found to characterise malignant melanocytic lesions in the MeLiM model and later in human melanomas, has also been found to be effective in distinguishing benign melanocytic tumours from melanomas in horses [105].

The complex inheritance of melanoma and coat/skin pigmentation (grey level, vitiligo grade, and speckling grade) in grey horses has been shown to be primarily due to the effects of a 4.6‐kb duplication in intron 6 of the *STX17 (*syntaxin 17) gene; this constitutes a *cis*‐acting regulatory mutation that has melanocyte‐specific effects [possibly due to the fact that it affects an enhancer that encodes binding sites for the microphthalmia‐associated transcription factor (MITF), which regulates melanocyte development] [106, 107]. Both *STX17* and the neighbouring *NR4A3* (*nuclear receptor subfamily 4, group A, member 3*) gene are overexpressed in melanomas from grey horses. Elevated *STX17* is associated with constitutive activation of the ERK pathway in melanocytic cells [108], thus highlighting the similarities to human melanoma in which the MAPK/ERK pathway is also involved. Similarly, just as melanocortin‐1 receptor (MC1R) plays an important role in regulating skin pigmentation and melanoma growth in humans and expression of agouti‐signalling protein (ASIP), a known MC1R antagonist, slows melanoma growth and increases survival times in mice [109], grey horses carrying a loss‐of‐function mutation in *ASIP* have a higher incidence of melanoma [106], suggesting that increased MC1R signalling promotes melanoma development in grey horses, as it does in humans.

**Figure 5:**
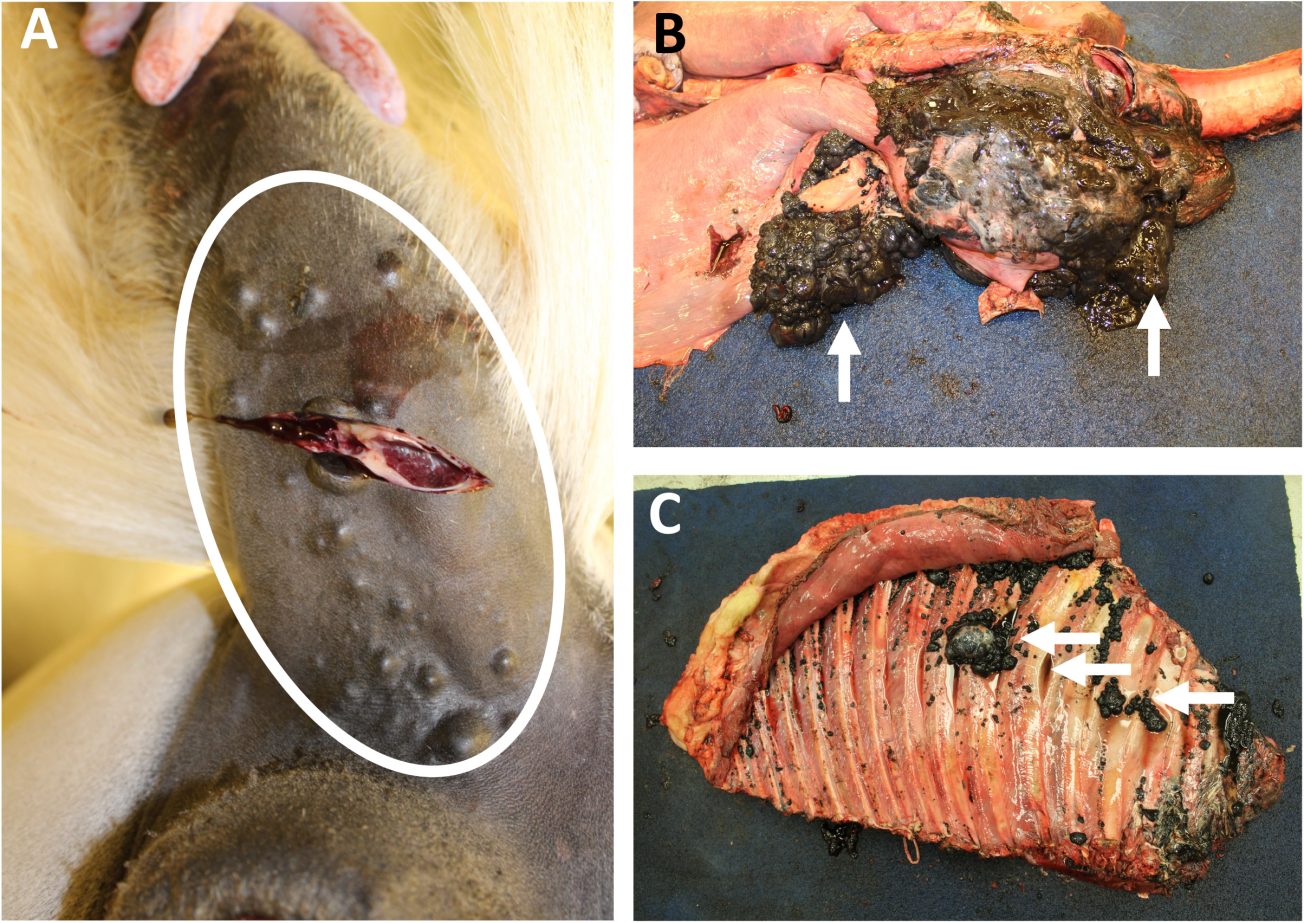
Metastatic melanoma in a horse. (A) Multiple melanomas located under the tail base (circle) that metastasised to the lungs and mediastinum around the heart. (B, C) Thoracic metastases (B; arrows) and metastases on the pleural surface of the ribs (C; arrows) are shown. The photographs were kindly provided by Laura Bassel, Department of Pathobiology, University of Guelph, Guelph, Ontario, Canada.

Melanoma nodules occur most frequently underneath the tail and at high rates in the perianal region, lips, and eyelids, with some also noted in the vulva [104, 110] (Figure 5). As many of these areas are near mucosal epithelium, equine melanomas may represent a model for mucosal melanoma in humans. Next‐generation sequencing of equine melanomas from mucosal‐like sites (perineum, perianal region, prepuce, vulva, or ventral tail), mucocutaneous sites (near the eyes or mouth, and showing both mucosal and haired skin), as well cutaneous sites (haired skin only) and other sites (urinary bladder wall muscle and the parotid gland) found that similar to human mucosal melanomas, most equine melanomas had less than five mutations per Mb [111], unlike UV‐associated human subtypes such as superficial spreading and nodular melanoma [112]. In addition, the most prominent driver genes were *NRAS* and *TP53*, as has been reported in human mucosal melanoma samples [111]. Both species also showed mutations in *PTEN* and *KIT* as well as a few cases with *BRAF* mutations. However, equine melanomas from mucosal‐like or mucocutaneous sites showed a landscape of driver genes that was less populated than human mucosal melanoma, and there were fewer recurrently mutated genes in common with human mucosal melanoma, with no mutations found in *SF3B1*, *ATRX* or *NF1* [111]. The equine cutaneous melanomas had no point mutations or indels in known human melanoma driver genes (apart from one having an *NRAS* ^*Q61R*^ mutation) [111], and as such they show some similarities to pigmented epithelioid melanocytoma (‘animal‐type melanoma’), a rare type of melanoma in humans that does not carry mutations in genes frequently mutated in cutaneous melanoma [113].

### Cat

**Figure 6:**
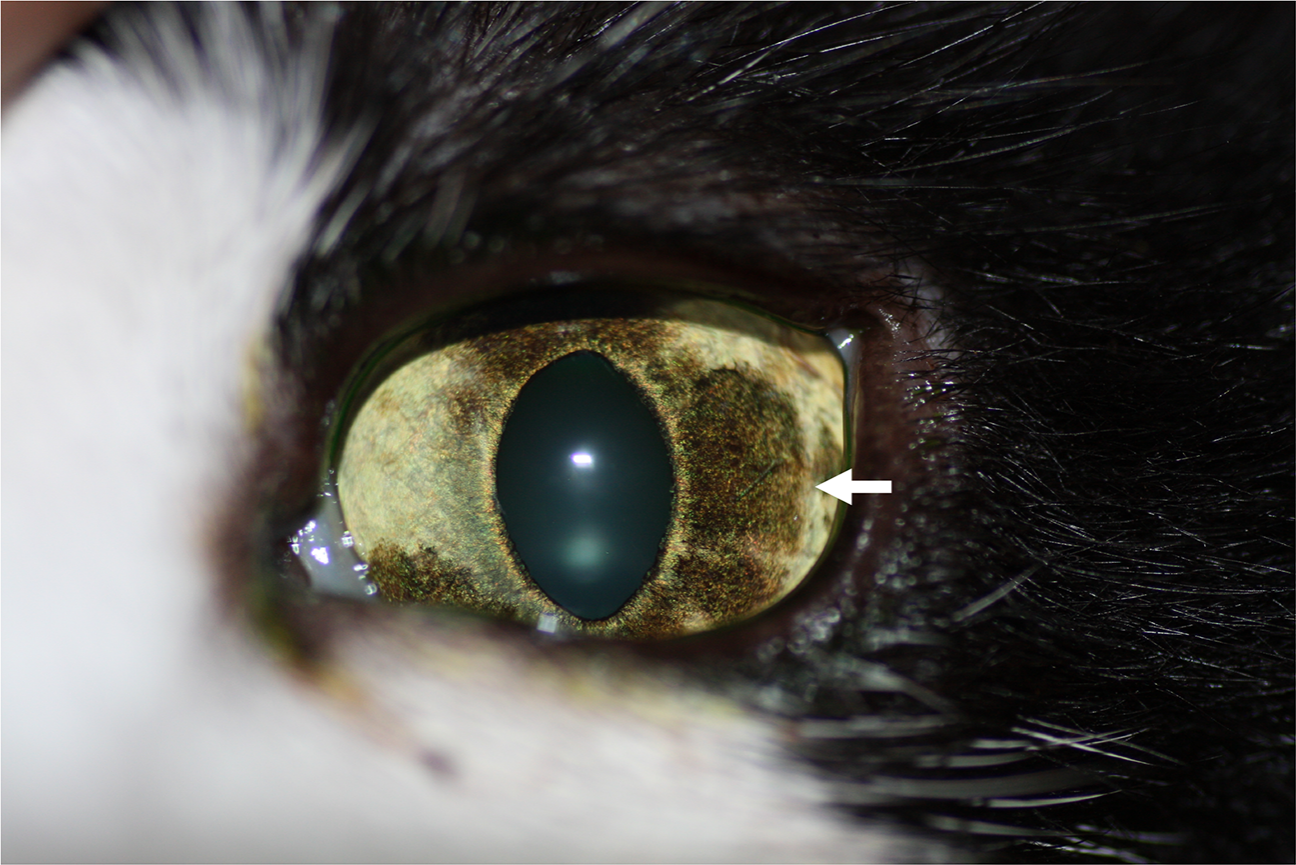
Uveal melanoma in a cat. Arrow indicates a diffuse iris melanoma. The photograph was kindly provided by Chantale Pinard, Department of Clinical Studies, University of Guelph, Guelph, Ontario, Canada.

Melanoma of any site in the cat is rare, with only four melanomas found out of 3,145 feline diagnoses at the Animal Medical Center in New York [114]. However, in contrast to other domesticated species such as dogs and horses, cats develop intraocular melanoma more frequently than cutaneous melanoma [115]. In a study of 29 feline melanomas over an 11‐year period, 19 melanomas were intraocular, with the iris being the most common area of involvement, metastasis occurring in 63% and 62.5% of the cats requiring euthanasia ~5 months post‐diagnosis [115]. Indeed, iris melanoma is the most frequent feline intraocular tumour (Figure 6), with only rare cases of choroidal melanoma being reported [116]. This is in contrast to humans, where most intraocular (uveal) melanomas originate in the choroid (with iris melanoma being rare); these do not usually spread and are associated with a greater than 95% 5‐year relative survival rate. Nevertheless, these feline ocular melanomas may offer an alternative to the other animal models of ocular melanoma, which suffer the limitations that they do not replicate the critical behaviour of the human disease, specifically spontaneous occurrence and concurrent metastasis (reviewed in [117]).

### Dog

Melanocytic tumours are relatively common in the dog, and are typically malignant; a study of 2,350 cases of melanocytic tumours found that 70% were malignant melanomas and 30% were benign tumours (melanocytomas) [118]. The anatomical locations are oral (62%), cutaneous (27%), digit (6%), ungual (4%), and ocular (1%) [118]. Interestingly, the correlation with malignancy varies at each site, with 84–100% of oral, digit, and ungual melanocytic tumours being malignant (melanoma), in contrast to only 43% and 29% of cutaneous and ocular melanocytic tumours, respectively [118].

#### Mucosal melanoma

**Figure 7:**
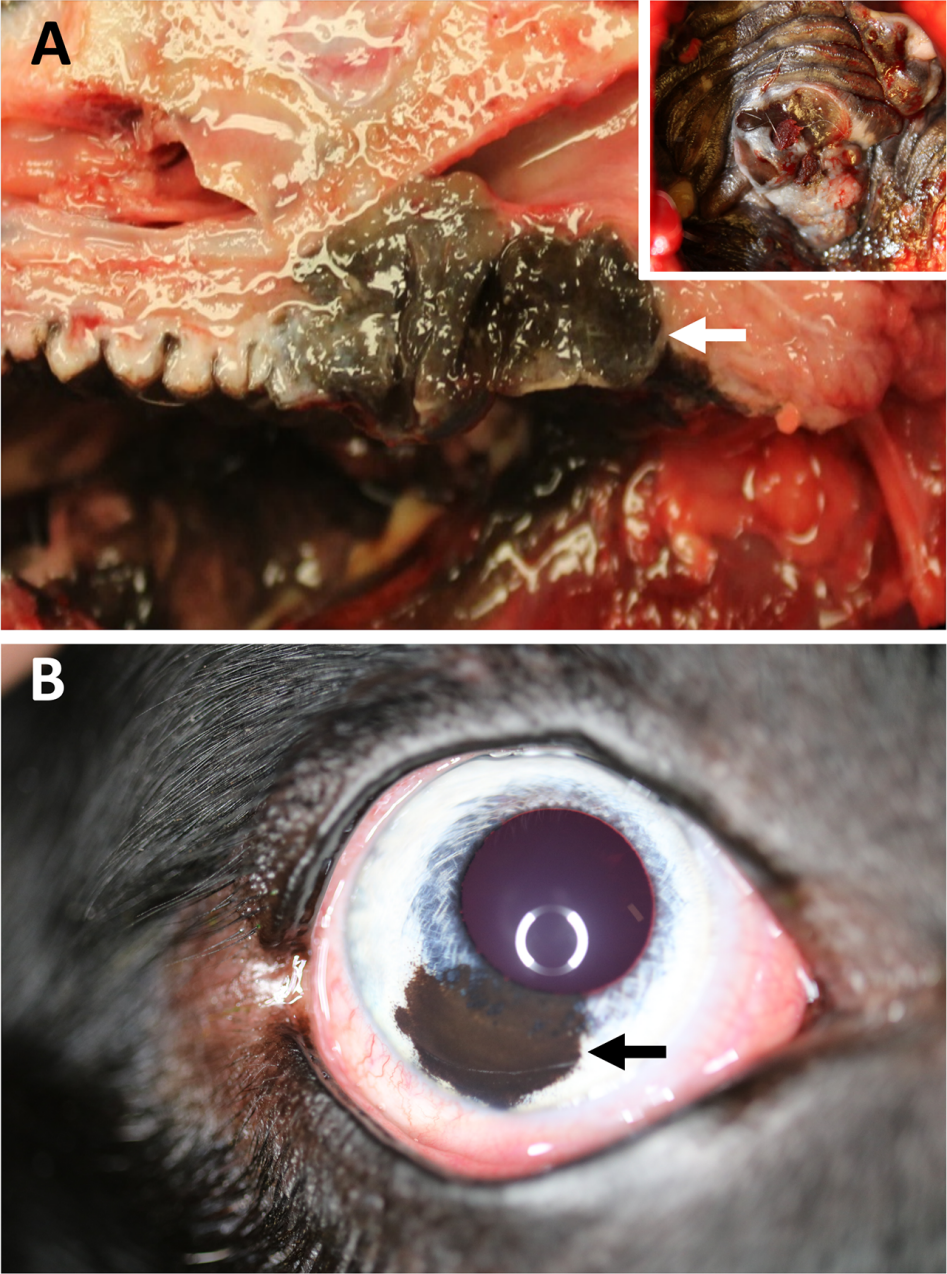
Oral and uveal melanomas in dogs. (A) A primary melanoma in the soft palate (inset) that invaded into the oropharynx (arrow). The photographs were kindly provided by Laura Bassel, Department of Pathobiology, University of Guelph, Guelph, Ontario, Canada. (B) A benign uveal melanoma (melanocytoma) in the iris (arrow). The photograph was kindly provided by Chantale Pinard, Department of Clinical Studies, University of Guelph, Guelph, Ontario, Canada.

Canine melanomas of the oral cavity occur mostly on the gingiva, but also on the lips, tongue, tonsils, palate, and oropharynx (Figure 7A) [118, 119]. Spontaneous anal sac, intestinal, and intranasal melanomas have also been reported, but these mucosal sites are rare [120]. Similar to humans, epidemiological studies have shown that certain breeds are more prone to develop oral melanoma, with Poodles, Golden Retrievers, Labradors, Rottweilers, and Yorkshire terriers representing 50% of the cases [120]. Similar to human mucosal melanoma, canine oral melanomas are aggressive, showing local invasiveness and a high propensity to metastasise to regional lymph nodes and lungs [121], with reported median survival times of 3–24 months (depending on stage and treatment) [118]. A further parallel with their human counterpart is that canine melanomas are resistant to chemotherapy and radiation therapy [122]. There are also similarities in response to therapy. Clinical trials showed that patients with metastatic mucosal melanoma can benefit from treatment with the immunotherapy regimes of nivolumab (targeted PD‐1) alone or in combination with ipilimumab (targeting CTLA‐4) [123] and similarly, a rat–dog chimaeric anti‐PD‐L1 monoclonal antibody was well tolerated and showed some clinical efficacy, with a response rate of 14.3% [124]. In addition, the use of human genes has also proved beneficial in treating canine oral melanoma. For example, in a phase II clinical trial using allogeneic whole‐cell vaccination, a canine melanoma cell line transfected with human *gp100*, killed by irradiation and intradermally administered to dogs with malignant melanoma, found objective evidence of tumour regression in 17.6% of cases, with dogs experiencing tumour control surviving significantly longer (337 days) than dogs having no response (95 days) [125]. Similarly, intratumoural administration of the human *CD94L* gene was evaluated in a phase I clinical trial in four dogs with oral melanoma and a 12–58% reduction of tumour burden was reported in three of the dogs, with no adverse effects observed [126]. Finally, a bacterial plasmid DNA vaccine encoding the human tyrosinase antigen (Oncept™) is licensed for adjuvant treatment of stage II and III canine oral melanoma after locoregional control and is currently awaiting USDA approval for use in horses with melanoma. However, it should be noted that there is controversy about its efficacy as some studies have observed responses in dogs with macroscopic disease and suggested that the vaccine could be considered as palliative treatment in stage IV disease [127], whilst others have found that dogs who received the vaccine did not achieve a greater progression‐free survival, disease‐free interval or median survival time than dogs that did not receive the vaccine [128].

A study that compared the histopathological features of human mucosal melanoma and canine oral melanoma found that analogous architectural features were important for diagnosis and staging in both species [129]. Interestingly, both human and canine mucosal melanomas included the range of epithelioid, spindloid, mixed epithelioid/spindloid, or small round blue cell melanocyte morphologies [129]. Also noteworthy was the frequent presence of a lentiginous intra‐epithelial component in canine melanomas, which is a feature known to precede invasive melanomas in humans that subsequently metastasise, thus indicating a similar pathway of progression in the dog [129].

At the molecular level, there are many similarities of canine oral melanoma with human mucosal melanoma. Next‐generation sequencing of canine oral melanomas revealed that they had less than five mutations per Mb, similar to human mucosal and equine mucosal melanomas [111]. In addition, the most prominent driver genes were *NRAS* and *TP53*, with both species also showing mutations in *NF1*, *PTEN*, and *KRAS* [111, 130]. Importantly, both species show involvement of the MAPK and PI3K/AKT pathways in mucosal melanoma formation and progression [111, 130, 131], with constitutive pathway activation and similar sensitivity to AZD6244 and rapamycin in human melanoma and canine oral melanoma cell lines [132], suggesting that canine oral melanoma could be a good model for human mucosal melanoma. However, mutations in other drivers of mucosal melanoma frequently seen in human patients, such as *SF3B1* and *ATRX*, were not seen, suggesting that these canine cancers may not represent a faithful model for the subset of human mucosal melanoma patients with mutations in these genes. Some canine oral melanomas showed mutations in *EIF1AX* (G9V and R13C), an essential translation initiation factor that is mutated in human uveal melanoma [8] and meningeal melanocytic tumours [133], with the same N‐terminal amino acid substitutions at conserved sites G9 and R13 being found in multiple other human tumours (COSMIC database: COSM6908971, COSM5899335) [111]; thus, canine oral melanomas have helped to identify the importance of these conserved regions in human tumourigenesis. In addition, cross‐species comparative copy number analysis found that the most significant deletion in human mucosal melanoma samples was a deletion at 15q15.1, which is syntenic to a deletion in canine oral melanomas found on canine chromosome 30; both species harbour *BUB1B*, *KNSTRN*, and *B2M* within this region and these genes are frequently altered in human tumours [111]. Similarly, both *MDM2* and *SMO* are amplified in human mucosal melanomas and canine oral melanomas, with these genes having known roles in cutaneous melanoma and a range of cancers [111]. In addition, a recent study of long non‐coding RNAs (lncRNAs) in canine oral melanomas found one downregulated (*SOX21‐AS*) and two upregulated (*CASC15* and *ZEB2‐AS*) lncRNAs (‘onco‐lncRNAs’) that are differentially expressed in a range of human cancers [134].

#### Cutaneous melanoma

Cutaneous melanocytic tumours in dogs are usually dermal in origin and are typically benign melanocytomas [135]. The most frequent sites for benign cutaneous melanomas are the face (near the eyelids), trunk, and extremities, and malignant melanoma is found most often on the head, ventral abdomen, and scrotum, with metastases primarily occurring to the lymph nodes and lungs [135]. Cutaneous melanoma occurs more commonly in dogs with heavily pigmented skin, with Schnauzers and Scottish Terriers at increased risk [136], which is in contrast to cutaneous melanoma in humans where individuals of light/fair skin are at increased risk.

There are limited data on somatic alterations of canine cutaneous melanoma. One study took a candidate gene approach with 20 cutaneous melanomas and found no variants mutations in the key melanoma drivers, specifically *BRAF*, *NRAS*, *PTEN*, *KIT*, *GNAQ*, and *CDK4* [118], whereas another study on two dogs found that one carried an *NRAS* mutation and one carried *KRAS*, *TP53*, and *KIT* mutations [130]. These reports, taken together with the fact that most canine cutaneous melanocytic tumours are benign and occur on hair‐bearing skin, thus are not linked to UV‐light exposure, suggest that these canine cutaneous melanomas arise from a distinct mechanism, different from that of most human cutaneous melanomas, and as such may be of more relevance to acral melanomas or other rare categories of dermal melanomas in humans [2].

#### Ocular melanoma

Melanocytic tumours are the most common ocular neoplasms in dogs, and arise most frequently in the anterior uvea; a study of intraocular melanocytic tumours in 244 dogs reported the anterior uvea as the tumour site in 85%, with the limbic area and choroid accounting for 13% and 5%, respectively [137]. Similar to cutaneous melanocytic tumours, most intraocular melanocytic tumours in dogs are benign (‘benign uveal melanoma’; Figure 7B), with reports of malignancy ranging from 15% to 30%, and those with metastatic behaviours accounting for ~4% [135]. The outcomes for dogs with histologically benign uveal melanoma is excellent; however, those with malignant uveal melanoma have worse outcomes and significantly shorter lifespans [137]. Interestingly, expression profiling using a 12‐gene assay reliably distinguished metastasising from non‐metastasising uveal melanomas in humans [138] and four of these genes (*HTR2B*, *FXR1*, *LTA4H*, and *CDH1*) are overexpressed in metastasising canine uveal melanoma [139].

#### Acral melanoma

Melanoma is the second most common digital tumour in dogs, accounting for 15–17% of neoplasias of the digits [140, 141]. Similar to human acral melanoma, which arises on the plantar surface of the foot, palms of hands, and fingers, canine acral melanoma arises on the footpad or nail bed. Most canine melanocytic tumours of the digits are malignant (49–86%) and all nail bed melanocytic tumours are malignant [118]. As with other forms of dog melanoma, the development of melanoma in the digits is over‐represented in some breeds, such as the Scottish Terrier, Schnauzer, Beauce Shepherds, and Rottweilers [140, 141]. These tumours are locally aggressive, with bone lysis occurring in 40–58% and have a high propensity to metastasise, with lymph node or lung metastasis found in 30–40% of cases at the time of presentation [140]. Only three melanomas from the digits of dogs have been characterised at the genomic level, with two having *KRAS* mutations and one an *NRAS* mutation [130].

#### Leptomeningeal melanoma

There has been little research into leptomeningeal melanoma/melanomatosis in dogs, most probably due to the rarity of the disease. Indeed, there is only one published report of primary CNS melanoma: an 11‐year old Black and Tan Coonhound with disseminated melanoma involving the right femoral bone marrow, lung, multiple lymph nodes, and adrenal gland, with diffuse infiltration of the leptomeninges of the brain and spinal cord. In light of the lesion distribution that resembled leptomeningeal melanomatosis in humans, the diagnosis was given as primary leptomeningeal melanomatosis with multi‐organ metastasis [142]. Similar to humans, secondary (metastatic) CNS melanoma is more common than primary CNS melanoma; post‐mortem studies of dogs with secondary intracranial tumours found them to be malignant melanomas in ~3.4% of cases [143, 144].

## Conclusion

The key feature(s) of each animal as a ‘model’ of melanoma in humans is/are listed in Table 3. No one animal is the perfect model. However, the spontaneous nature of melanoma development and its metastatic spread in the platyfish/swordtail, opossum, miniature pig, horse, cat, and dog, together with the genetic heterogeneity of the latter three species and the additional advantage of shared microenvironmental exposures in companion animals, surely make these animals attractive candidate models.

**Table 3:** Key features of each animal as a ‘model’ of melanoma in humans.

Animal	Key features as a model
Platyfish/swordtail	Melanoma arises from the EGFR pathway (uses Ras/Raf/MAPK signalling) Melanoma incidence controlled by a pigment‐cell‐specific oncogene locus and a tumour suppressor locus Model of UV radiation‐induced melanoma
Gray short‐tailed opossum	Model of UV radiation‐induced melanoma Model of sunscreen‐mediated melanoma prevention
Miniature pig	Opportunity to study the entire spectrum of melanoma formation, from benign lesions to malignant transformation to metastatic spread Model of spontaneous regression of melanomas
Horse	Melanomas under the tail and in the perianal region; lips and eyelids are models of mucosal melanoma Driver genes in these mucosal melanomas: *NRAS*, *TP53*, *PTEN*, *KIT*, and *BRAF*
Cat	Melanomas of the uvea are a model of uveal melanoma
Dog	Melanomas of the oral cavity are a model of mucosal melanoma Driver genes in these mucosal melanomas: *NRAS*, *TP53*, *PTEN*, *NF1*, and *KRAS* MAPK and PI3K/AKT pathways involved in mucosal melanoma formation and progression Melanomas of the footpad or nail bed are models of acral melanoma Clinical trials are performed in dogs with melanoma

## Author contributions statement

LvdW and TB wrote the genetics and pathology of human melanoma section. LvdW, EEP and GAW wrote the sections on the occurrence of melanoma in animals. EEP, GAW and DJA coordinated the photographs. All the authors contributed to revision of the manuscript and the final published paper. All the authors contributed equally.
